# Assessing and improving communication strategies among healthcare providers and African American individuals with systemic lupus: a scoping review

**DOI:** 10.1186/s41927-026-00665-5

**Published:** 2026-06-26

**Authors:** Mia L. Barron, Robin M. Dawson, Cynthia F. Corbett, Michael D. Wirth, Edith M. Williams

**Affiliations:** 1 School of Nursing, University of South Carolina, 1601 Greene Street, Columbia, SC 29209 USA; 2 Center for Community Health & Prevention, University of Rochester, 46 Prince Street, Rochester, NY 14607 USA

**Keywords:** Systemic lupus erythematosus, SLE, Lupus, Communication, Professional patient relations, African Americans

## Abstract

**Background:**

Systemic Lupus Erythematosus (SLE) is a severe autoimmune disorder that impacts multiple organ systems, with African American (AA) adults having worse outcomes and poor prognoses compared to other ethnic groups. Ineffective patient–provider communication and inadequate screening for modifiable risk factors contribute to poorer SLE outcomes and ongoing health disparities. This scoping review evaluated the influence of patient–provider communication among adults with SLE, with particular attention to implications for African American populations, to inform the development of strategies to mitigate SLE health disparities.

**Methods:**

An electronic search was conducted using PubMed, CINAHL, Embase and PsycINFO databases to systematically identify published literature between 2012 and mid-2025. Studies were eligible (1) if participants included African American adults (over 18 years old) with a diagnosis of SLE or included a sample with ≥ 50% African American representation, (2) were published in the English language, (3) reported databased original research results, and (4) reported findings related to patient-provider communication, a communication tool, or a communication strategy either independently or in conjunction with health outcomes.

**Results:**

Search results yielded **214** articles. After an in-depth screening process using the PRISMA method, **20** articles met both inclusion and exclusion criteria: seven qualitative, six quantitative, and seven mixed methods. Two studies enrolled exclusively African American participants. A central theme was the importance of embedding effective communication strategies into evidence-based decision aids for individuals with SLE. Additional priorities included identifying key facilitators of communication, such as family involvement, slower speech, and empathy, followed by the need to validate patient-reported outcome measures that support better information exchange. Nevertheless, none of the studies examined targeted communication tools aimed at reducing modifiable risk factors or improving health outcomes among African Americans with SLE, indicating a critical gap warranting further investigation.

**Conclusions:**

Research on effective patient-provider communication with African American adults living with SLE is limited and lacks evaluation of specific communication strategies. Findings demonstrated that effective communication, marked by trust, clarity, and adequate appointment duration positively influence the clinical experiences of African American adults with SLE.

**Clinical trial number:**

Not applicable.

## Background

Systemic lupus erythematosus (SLE) is a severe chronic autoimmune disease that affects multiple organ systems and is frequently associated with significant morbidity and disability [1]. In the United States, SLE disproportionately affects African American (AA) adults, who experience higher incidence, earlier disease onset, and worse clinical outcomes compared to other racial and ethnic groups [1, 2]. Despite advances in treatment and improved survival rates, AA adults continue to experience poorer prognoses, highlighting persistent disparities in SLE outcomes.

While prior research has extensively examined the clinical manifestations and disease burden of systemic lupus erythematosus (SLE), limited attention has been given to the role of modifiable factors, such as patient–provider communication, in shaping health outcomes [1, 3]. Although direct evidence remains limited, existing findings suggest that effective patient–provider communication may improve patient well-being, treatment adherence, and engagement with evidence-based care standards [4]. In addition, traditional approaches to assessing disease burden often rely on clinical metrics and standardized measures that may not fully capture patients’ lived experiences or communication needs. Misalignment between patient and provider perspectives, particularly regarding symptom prioritization, perceptions of disease impact, and responsiveness to patient concerns may contribute to mistrust, poor adherence, and suboptimal outcomes [4]. Although communication in the management of other chronic conditions affecting minority populations such as cancer [5], diabetes [6], HIV [7], and sickle cell disease [8] has been explored, research on SLE in AA adults remains limited.

Therefore, the purpose of this scoping review is to evaluate the influence of patient–provider communication among adults with SLE, with an emphasis on factors that may differentially impact AA individuals to inform the development of effective strategies to mitigate SLE health disparities. Specifically, this review examines evidence related to communication practices, tools, and strategies and their association with health outcomes, including symptom management, self-management behaviors, and healthcare utilization. Additionally, this review aims to identify gaps in the literature, particularly the lack of targeted communication interventions addressing modifiable risk factors among AA adults with SLE.

## Methods

### Search strategy

A scoping review was conducted using four databases: CINAHL, PubMed-Medline, Embase, and PsycINFO. These databases were chosen for optimum retrieval of studies that included both clinical and sociobehavioral aspects of care, which are essential for comprehending communication and inequities in SLE. To identify SLE terms, we used the MeSH database. To identify race/ethnicity terms, we slightly modified a search strategy that was used in a systematic review examining racial/ethnic disparities about adherence to surveillance mammograms among breast cancer survivors [9]. We included general terms on race and ethnicity groups (race, ethnicity, minorities) and specific race and ethnicity groups (e.g., African Americans, Blacks). Initially, only two studies exclusively involved AA participants; thus, studies incorporating participants from all racial and ethnic backgrounds were also considered, as long as AA participants were represented in the sample or constituted at least 50% of the study population, in accordance with the review eligibility criteria. This method was significant due to research indicating that minority communities, especially AA individuals, endure a disproportionate burden of systemic lupus erythematosus [10]. To identify patient-provider communication terms, the search strategy was modeled after a recent systematic review examining physician-patient communication and its influence on health outcomes in rheumatology [11]. Terms such as provider-patient communication, provider-patient relationship, and physician-patient interactions were included in the search (Table 1). When combining these search strategies, overlapping search terms were identified and removed. For completeness, bibliographies of selected studies were systematically searched to capture additional relevant cited articles published from 2012-mid-2025. Search strategies, including the search filters, terms used, and the number of articles retrieved from each database, are listed in Table 1.

### Inclusion criteria

Studies including diverse populations were eligible, however, studies were required to include AA participants or report findings specific to AA participants. Studies lacking demographic reporting were noted as a limitation in assessing equity relevance. Additionally, only studies directly related to patient-provider communication and SLE were considered to ensure that the selected literature aligned with the goals and objectives of the review findings. All study designs (i.e., qualitative, quantitative, or mixed methods study design) were considered eligible for review if the study was peer reviewed, published in English, and the purpose included examining patient-provider communication and/or a communication tool or strategy for adults (≥ 18 years) with SLE. The search was limited to a 13-year time period between 2012 and 2025 in order to evaluate the ever-evolving, current research, practices, policies, and guidelines that influence SLE outcomes and to best capture contemporary communication strategies such as digital and electronic communication health tools (e.g. EHR, MyChart), cultural and linguistic competences, trauma and emotional health, implicit bias, and racism.

### Exclusion criteria

Studies in the form of non-peer-reviewed articles such as abstracts, editorials, commentary, dissertations, grey literature, and topics unrelated to communication and SLE were excluded from the review due to concerns about the quality and reliability of the data.

### Screening process and data extraction

**Fig. 1:**
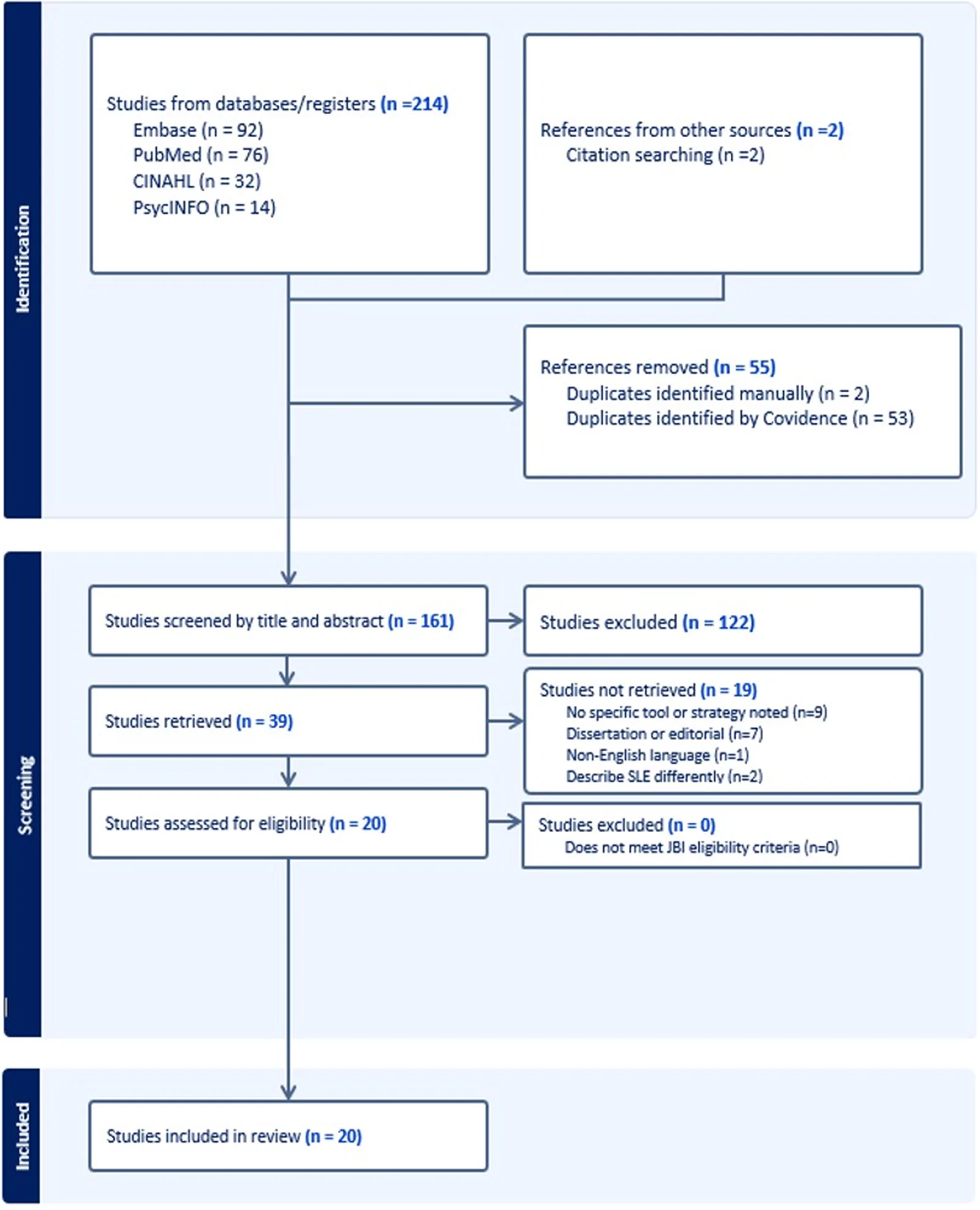
Prisma flow diagram of literature review results

All identified records were imported into the Covidence citation management tool and duplicates were removed. Title and abstract screening were conducted independently using predefined eligibility criteria. Full-text articles were then assessed for inclusion by the same reviewer. Discrepancies at any stage were resolved through discussion and consensus with consultation from a second reviewer. A standardized data extraction template was created for collating data from each record to include the following headings: study year/author, purpose, study design/sample size (racial/ethnic distribution), and findings (see Table 2). The search results from all four databases and citations yielded 214 articles, with 55 duplicates manually removed from the Covidence citation management tool. Titles and abstracts of the remaining articles were screened for eligibility and inclusion. A total of 121 articles were excluded following the title and abstract review due to being irrelevant, and one article could not be retrieved. Full-text review of the remaining 39 articles resulted in 19 exclusions. Of the excluded articles, two used the SLE acronym for different conditions unrelated to systemic lupus erythematosus (e.g. specialized learning experiences). Also, among the excluded articles, nine were either unrelated to the content area and/or did not describe a specific communication tool or strategy. One was written in non-English language while seven were described as either a dissertation or an editorial. The methodological quality was evaluated utilizing the Joanna Briggs Institute (JBI) critical appraisal methods pertinent to each study design. Twenty articles were analyzed using the Joanna Briggs Institute Critical Appraisal Guidelines [12]. The appraisal outcomes were not utilized to exclude investigations; instead, they were employed to guide the interpretation of findings and to contextualize the quality of the evidence. Additionally, the appraisal results informed the application through an equity lens by highlighting deficiencies in the inclusion and reporting of AA participants and aiding in the identification of evidence gaps related to inequities. Of the 20 articles, communication tools, program models, or strategies to improve outcomes among patients with SLE and the potential influence on SLE disparities were described in all articles (see Fig. 1). The findings were summarized narratively via thematic analysis methodologies. Studies relating to communication were coded inductively, exhibiting recurring concepts across research regardless of the methodological approach. Codes were thoroughly investigated and grouped into major thematic groups that reflect common trends in the literature.

## Results

### Study characteristics

The studies included in this review represented a combination of qualitative (*n* = 7), quantitative (*n* = 6), and mixed-methods (*n* = 7) designs, providing a diverse methodological foundation for understanding patient–provider communication in SLE. Consistent with the study designs, the body of evidence demonstrated wide variability in approach and sample size, with participant numbers ranging from 23 to 698. Most study participants were female, reflecting the demographics of the broader lupus population. Additionally, over half (68%) of the 20 selected articles featured predominantly AA samples, consistent with the disproportionate prevalence of SLE within this group. Across study designs, mistrust and inadequate communication as obstacles to care were consistent findings, revealing relationships between the quality of communication, treatment adherence, and health outcomes. Results from the qualitative and mixed-methods study designs reinforced these findings by emphasizing patients’ perceptions of being unheard, disregarded, or inadequately supported during clinical interactions.

Several themes emerged related to patient–provider communication, most notably the need to develop, test, and refine effective strategies for implementing evidence-based shared decision-making tools tailored to individuals with SLE. Validated patient-reported outcome measures to enhance information transmission are also needed. Priority areas for improving practice included the identification of essential communication facilitators, namely, family participation, clear and measured communication, and empathy. Table 3 provides an evidence map summarizing the design, population, communication domains, outcome measures, and principal findings of the included studies.

### Shared decision-making tools in SLE: development and implementation gaps

A primary theme discovered was the incorporation of effective communication tactics into evidence-based decision aids for patients with systemic lupus erythematosus (SLE). Evidence suggests that model programs and/or shared decision-making tools may improve patient-centered communication [13–15], care [16] and outcomes [17, 18]. In one study, AA women who participated in the Chronic Disease Self-Management Program (CDSMP) reported feeling better prepared to participate in self-management behaviors and communicate with their physicians, suggesting the CDSMP may be an effective tool for enhancing patients’ communication skills [19, 20].

The Lupus Impact Tracker (LIT) was also deemed an appropriate tool used in the clinical practice setting to evaluate the effects of lupus on patients’ daily activities [13]. Most importantly, the LIT obtained distinct information absent from the conventional physician disease assessments and may facilitate patient-provider communication from the patient’s perspective. However, participants in the longitudinal study were mostly White.

The SMILE (Shared Decision-Making in Lupus Electronic) tool represents a comprehensive approach to supporting treatment decisions and was shown to reduce decisional conflict [14]. Healthcare providers and patient advocacy leaders perceived the SMILE tool as promising for enhancing patient-provider communication and quality improvement. Nonetheless, numerous obstacles were encountered during SMILE implementation, including patient-related factors (time limitations, health literacy), clinic-related factors (staff workload, spatial constraints), technology-related factors (Wi-Fi connectivity, iPad navigation), and management-related factors (workflow integration, necessity for advocates).

The LFA SELF (Strategies to Embrace Living with Lupus Fearlessly) web-based program represents a significant advancement in lupus self-management education [15]. This online program was created based on the Transtheoretical Model of Behavior Change and emphasizes four principal self-management strategies: symptom management, stress management, medication management, and collaboration with healthcare providers. Pilot study findings revealed that over 80% of participants had moderate or high skill gaps at program admission, while approximately 60% of those who completed the program mastered one or more self-management skills. The program impact on patient-provider interactions and fatigue interference was significant. However, the findings must be interpreted cautiously due to its small, non-randomized study sample and the potential for selection bias, which restricts generalizability and clinical usefulness from a patient-provider communication perspective. Individuals with low literacy, visual impairments, or limited technology availability may also be restricted from accessing LFA SELF, hindering the equitable enhancement of communication across diverse patient populations.

### Key facilitators of effective patient–provider communication

A prominent pattern discerned in the reviewed research was the significance of essential communication facilitators specifically family engagement, coherent and measured communication, and empathy in improving patient-provider relations in SLE. Evidence indicates that poor communication results in lower self-management and greater disease severity [1, 3, 21]. In qualitative studies, AA participants with SLE noted that clinic staff lacked empathy and understanding of the SLE experience [22], and medically underserved patients identified the need for better communication at diagnosis and better navigation throughout treatment and care [23, 24]. Relatedly, in another study, the adjusted interpersonal processes of care value for rushed dialogue, explanation outcomes, and rude office personnel scales were all substantially linked to depressive symptom severity [3]. Two studies provided evidence that family involvement, empathy, slower speech, and avoiding difficult words promote effective patient-provider communication [1, 22].

### Role of patient-reported outcome measures in enhancing information exchange

A major theme uncovered from the included research was the significance of validated patient reported outcomes measures (PROs) to improve communication and information exchange in SLE. Commonly used PROs in SLE include both disease-specific measures, such as SLAQ, LupQoL, and generic instruments such as the SF-36 and PROMIS. While these tools capture important aspects of disease activity and quality of life, their ability to fully reflect patient priorities and support effective patient–provider communication remains variable [13]. Additionally, PROs assessments tools such as Patient Global Assessment (PtGA) and others used to assess and examine the impact of SLE on patients’ physical functioning, mental, and social well-being among patients with SLE indicated elevated levels of comprehension, utility, and acceptability, particularly within a large AA demographic [18, 25, 26]. Most participants perceived the tailored functioning reports as comprehensible (70.2–85.1% rated them very easy) and beneficial for treatment planning (70.2–80.5%). Almost all participants expressed comfort in discussing these reports with healthcare providers (93.2–100%) [18]. However, methods of assessing disease burden for SLE frequently fail to fully encompass the wide range of difficulties and effects patients encounter and may not fully capture the comprehensive picture of managing a chronic autoimmune condition like SLE [13]. Further, measures that are used more often reflect those important to providers and clinical management rather than aspects of concern for patients with SLE. For example, patients may not consider certain physical SLE symptoms like rashes or alopecia as important, or they may attribute these symptoms to other factors. In contrast, healthcare providers often view these physical indicators of disease activity as significant [27]. At the same time, experiential symptoms (e.g., pain, fatigue) can only be self-reported by the patient but are often diminished or even discounted by providers [13]. These differing perspectives and priorities related to SLE disease management can result in patient distrust, dissatisfaction, treatment nonadherence, interruptions, delays, and, ultimately, negatively affect patient outcomes [28]. Furthermore, their integration into the routine clinical communication and decision-making processes remains limited. Validated PROs have the potential to enhance patient engagement and comprehension of their care, support shared decision-making, and improve information sharing between patients and providers when implemented effectively.

### Equity considerations and racial representation

Interestingly, in addition to specific tools, models and strategies for improving patient provider communication, most recent studies have shown that women of color with autoimmune diseases must undertake substantial efforts to establish credibility in order to be treated seriously by healthcare professionals [29]. Required efforts include racial framing for context, code-switching to align with White linguistic standards and recruiting credible witnesses. Women of color encounter dual dilemmas as patients wherein authenticity may lead to discrimination, while concealing elements of their identity can result in compromised care [29]. Additionally, the findings from Yalavarthi and colleagues [24] suggest that AA adults’ experiences of racism and social risk factors may influence therapy and outcomes. Similarly, Rodgers’ and others ethnography results identified that marginalized populations’ healthcare perceptions and decision-making are critical to developing culturally tailored interventions to improve patient outcomes and reduce disparities [30]. That finding was supported by those of a mixed methods study in which researchers examined racial ethnic differences between AA and White adults with SLE and suggested education about therapy can improve patients’ trust in the providers and influence treatment decisions [31].

## Discussion

In this scoping review, we examined communication tools and strategies aimed at improving patient–provider communication and outcomes among adults with SLE. Most studies evaluated general communication interventions; however, none assessed tools specifically tailored to AA adults. Despite the disproportionate burden of SLE in this population, few studies explicitly examined communication within AA groups, highlighting a critical knowledge gap.

The available evidence suggests that patient–provider communication may influence treatment adherence and care experiences, although findings remain limited and heterogeneous [32]. Ineffective communication has been associated with diagnostic delays, missed appointments, and poor medication adherence [33], and may inadequately address patients’ concerns and informational needs [27]. Broader factors, including social determinants of health such as structural racism and limited access to care, further contribute to disparities, with some evidence suggesting communication may play a role in mitigating these effects [34].

Although prior studies indicate that interventions aimed at improving patient–provider communication may reduce discordance, improve adherence, and enhance disease outcomes [32, 35, 36], there is a lack of evidence evaluating standardized or targeted communication strategies for AA adults with SLE. These findings highlight the potential importance of communication as a modifiable factor in addressing disparities, while emphasizing the need for more rigorous and inclusive research. Future studies should prioritize the development and evaluation of culturally responsive communication tools and strategies, as well as comparative analyses across diverse racial and ethnic populations, to better understand both shared and population-specific contributors to disparities in SLE outcomes.

### Limitations

There are several limitations to this scoping review. Articles described minimal to no specific communication strategies used among AA adults with SLE and their providers. Although other racial and ethnic groups were mentioned in the selected articles, in this literature review we exclusively focused on one ethnic group, which could introduce potential bias in the findings and interpretations. Several tools were mentioned, but none of the researchers provided evidence of reliability or validity for measuring effective patient-provider communication specific to AA adults. Additionally, variability in study design, patient populations, and measurement instruments created a lack of clarity and consistency in defining what constitutes “effective” patient–provider communication, making it difficult to operationalize within the research evidence. Furthermore, only a limited number of articles addressed the lived experiences of adults with SLE or explored potential racial and culturally tailored interventions.

## Conclusion

AA adults with SLE in the US experience higher mortality rates than White adults [28, 37]. Although survival has improved, early mortality persists and is associated with disease severity and modifiable risk factors. Despite this disproportionate burden, AA patients remain underrepresented in the literature, and few studies examine patient–provider communication within this population. Available evidence indicates that communication barriers, including mistrust and limited patient-centered responsiveness, are associated with disparities in care and outcomes. While SLE management focuses on controlling disease activity and preventing organ damage, differences in patient and provider perspectives may contribute to communication gaps that affect adherence and self-management, particularly in the context of broader disparities such as limited access to care and higher disease burden. Communication strategies are inconsistently defined and evaluated, and their independent impact remains unclear due to methodological variability and predominantly small studies. These findings highlight important gaps and the need for research evaluating culturally responsive communication approaches in SLE, particularly among AA adults.

**Table 1:** Search summary

Database Search Summary			
Database	Search Filters	Search Terms	Citations Returned
PubMed	English	((“lupus erythematosus, systemic“[MeSH Terms] OR “lupus“[Title/Abstract] OR “SLE“[Title/Abstract]) AND (“decision making, shared“[MeSH Terms] OR “Professional-Patient Relations“[MeSH Terms: noexp] OR “Physician-Patient Relations“[MeSH Terms] OR “Trust“[MeSH Terms] OR “Empathy“[MeSH Terms] OR “Patient-Centered Care“[MeSH Terms] OR “Communication“[MeSH Terms] OR (“clinician patient relation*“[Title/Abstract] OR “clinician patient interact*“[Title/Abstract] OR “communicat*“[Title/Abstract] OR “doctor patient relation*“[Title/Abstract] OR “doctor patient interact*“[Title/Abstract] OR “decision making“[Title/Abstract] OR “medical relation*“[Title/Abstract] OR “medical interact*“[Title/Abstract] OR “patient clinician relation*“[Title/Abstract] OR “patient clinician interact*“[Title/Abstract] OR “patient doctor relation*“[Title/Abstract] OR “patient doctor interact*“[Title/Abstract] OR “patient provider relation*“[Title/Abstract] OR “patient provider interact*“[Title/Abstract] OR “patient rheumatologist“[Title/Abstract] OR “rheumatologist patient“[Title/Abstract] OR “physician patient relation*“[Title/Abstract] OR “physician patient interact*“[Title/Abstract] OR “provider patient relation*“[Title/Abstract] OR “provider patient interact*“[Title/Abstract] OR “empath*“[Title/Abstract] OR “listen*“[Title/Abstract] OR “Patient-Centered Care“[Title/Abstract] OR “relationship centered care“[Title/Abstract] OR “shared decision“[Title/Abstract] OR “trust*“[Title/Abstract])) AND (“Black or African American“[MeSH Terms] OR “Ethnic and Racial Minorities“[MeSH Terms] OR “Health Status Disparities“[MeSH Terms] OR “Race Factors“[MeSH Terms] OR “Minority Groups“[MeSH Terms] OR (“african american*“[Title/Abstract] OR “Black“[Title/Abstract] OR “Blacks“[Title/Abstract] OR “disparit*“[Title/Abstract] OR “men of color“[Title/Abstract] OR “minorit*“[Title/Abstract] OR “people of color“[Title/Abstract] OR “race“[Title/Abstract] OR “racial*“[Title/Abstract] OR “women of color“[Title/Abstract]))) AND (2012/1/1:2025/1/3[pdat])	76
CINAHL	English	(MH “Lupus Erythematosus, Systemic+” OR TI (SLE OR lupus) OR AB (SLE OR lupus)) AND ((MH “Decision Making, Shared” OR MH “Professional-Patient Relations” OR MH “Physician-Patient Relations” OR MH “Trust” OR MH “Empathy” OR MH “Patient Centered Care” OR (MH “Communication " OR MH “Nonverbal Communication " OR MH “Communication Protocols " OR MH “Communication Skills” OR MH “Communication Barriers " OR MH “Persuasive Communication” OR MH “Scholarly Communication” OR MH “Communication Methods, Total” OR MH “Alternative and Augmentative Communication” OR MH “Communication Skills Training”)) OR TI (“clinician patient relation*” OR “clinician patient interact*” OR communicat* OR “decision making” OR “doctor patient relation*” OR “doctor patient interact*” OR empath* OR listen* OR “medical interact*” OR “medical relation*” OR “patient centered care” OR “patient clinician relation*” OR “patient clinician interact*” OR “patient doctor relation*” OR “patient doctor interact*” OR “patient provider relation*” OR “patient provider interact*” OR “patient rheumatologist” OR “physician patient relation*” OR “physician patient interact*” OR “provider patient relation*” OR “provider patient interact*” OR “relationship centered care” OR “rheumatologist patient*” OR “shared decision” OR trust*) OR AB (“clinician patient relation*” OR “clinician patient interact*” OR communicat* OR “decision making” OR “doctor patient relation*” OR “doctor patient interact*” OR empath* OR listen* OR “medical interact*” OR “medical relation*” OR “patient centered care” OR “patient clinician relation*” OR “patient clinician interact*” OR “patient doctor relation*” OR “patient doctor interact*” OR “patient provider relation*” OR “patient provider interact*” OR “patient rheumatologist” OR “physician patient relation*” OR “physician patient interact*” OR “provider patient relation*” OR “provider patient interact*” OR “relationship centered care” OR “rheumatologist patient*” OR “shared decision” OR trust*)) AND (MH “African Americans” OR (MH “Minority Groups+” OR MH “Ethnic Groups+”) OR MH “Health Status Disparities+” OR MH “Race Factors” OR TI (“African American*” OR Black OR Blacks OR disparit* OR “men of color” OR minorit* OR “people of color” OR race OR racial* OR “women of color”) OR AB (“African American*” OR Black OR Blacks OR disparit* OR “men of color” OR minorit* OR “people of color” OR race OR racial* OR “women of color”))	32
PsycINFO	English	(DE “Lupus” OR TI (SLE OR Lupus) OR AB (SLE OR Lupus)) AND (DE “Trust (Social Behavior)” OR DE “Empathy” OR (DE “Communication” OR DE “Augmentative Communication” OR DE “Communication Barriers” OR DE “Electronic Communication” OR DE “Humor” OR DE “Interpersonal Communication” OR DE “Messages” OR DE “Nonverbal Communication” OR DE “Organizational Communication” OR DE “Persuasive Communication” OR DE “Privileged Communication” OR DE “Scientific Communication” OR DE “Semiotics” OR DE “Social Communication” OR DE “Terminology” OR DE “Verbal Communication”) OR (DE “Therapeutic Processes” OR DE “Dual Relationships”) OR TI (“clinician patient relation*” OR “clinician patient interact*” OR communicat* OR “decision making” OR “doctor patient relation*” OR “doctor patient interact*” OR empath* OR listen* OR “medical interact*” OR “medical relation*” OR “patient centered care” OR “patient clinician relation*” OR “patient clinician interact*” OR “patient doctor relation*” OR “patient doctor interact*” OR “patient provider relation*” OR “patient provider interact*” OR “patient rheumatologist” OR “physician patient relation*” OR “physician patient interact*” OR “provider patient relation*” OR “provider patient interact*” OR “relationship centered care” OR “rheumatologist patient*” OR “shared decision” OR trust*) OR AB (“clinician patient relation*” OR “clinician patient interact*” OR communicat* OR “decision making” OR “doctor patient relation*” OR “doctor patient interact*” OR empath* OR listen* OR “medical interact*” OR “medical relation*” OR “patient centered care” OR “patient clinician relation*” OR “patient clinician interact*” OR “patient doctor relation*” OR “patient doctor interact*” OR “patient provider relation*” OR “patient provider interact*” OR “patient rheumatologist” OR “physician patient relation*” OR “physician patient interact*” OR “provider patient relation*” OR “provider patient interact*” OR “relationship centered care” OR “rheumatologist patient*” OR “shared decision” OR trust*)) AND (DE “Black People” OR (DE “Minority Groups” OR DE “Racial and Ethnic Groups” OR DE “African Cultural Groups” OR DE “Black People” OR DE “People of Color”) OR DE “Health Disparities” OR TI (“African American*” OR Black OR Blacks OR disparit* OR “men of color” OR minorit* OR “people of color” OR race OR racial* OR “women of color”) OR AB (“African American*” OR Black OR Blacks OR disparit* OR “men of color” OR minorit* OR “people of color” OR race OR racial* OR “women of color”))	14
Embase	English	(‘systemic lupus erythematosus’/exp OR lupus: ti, ab OR sle: ti, ab) AND (‘professional-patient relationship’/de OR ’doctor patient relationship’/exp OR ’trust’/exp OR ’empathy’/exp OR ’interpersonal communication’/exp OR ’clinician patient relation*’:ti, ab OR ’clinician patient interact*’:ti, ab OR ’doctor patient relation*’:ti, ab OR ’doctor patient interact*’:ti, ab OR ’medical relation*’:ti, ab OR ’medical interact*’:ti, ab OR ’patient clinician relation*’:ti, ab OR ’patient clinician interact*’:ti, ab OR ’patient doctor relation*’:ti, ab OR ’patient doctor interact*’:ti, ab OR ’patient provider relation*’:ti, ab OR ’patient provider interact*’:ti, ab OR ’patient rheumatologist’:ti, ab OR ’physician patient relation*’:ti, ab OR ’physician patient interact*’:ti, ab OR ’provider patient relation*’:ti, ab OR ’provider patient interact*’:ti, ab OR ’rheumatologist patient’:ti, ab OR communicat*:ti, ab OR empath*:ti, ab OR listen*:ti, ab OR ’patient centered care’:ti, ab OR ’relationship centered care’:ti, ab OR ’shared decision’:ti, ab OR trust*:ti, ab) AND (‘black person’/de OR ’african american’/exp OR ’race’/exp OR ’health disparity’/exp OR ’minority group’/exp OR black: ti, ab OR blacks: ti, ab OR ’african american*’:ti, ab OR disparit*:ti, ab OR ’men of color’:ti, ab OR minorit*:ti, ab OR ’people of color’:ti, ab OR race: ti, ab OR racial*:ti, ab OR ’women of color’:ti, ab) AND [2012–2025]/py (‘article’/it OR ’article in press’/it OR ’review’/it) AND ([adult]/lim OR [aged]/lim OR [middle aged]/lim OR [very elderly]/lim OR [young adult]/lim)	92

**Table 2:** Summary of retrieved articles

Year/authors	Purpose	Study design	SAMPLE/population	Outcomes	KEY findings
2012/ Drenkard, et al. [20]	To evaluate the feasibility and preliminary effectiveness of the Chronic Disease Self-Management Program designed to improve health outcomes and disease management among low-income African American women with systemic lupus erythematosus (SLE).	Quantitative Descriptive; **pilot quasi-experimental study** using a **single-group pre–post intervention design** (no control group)	45 female participants/Mean Age: 43.6 Low-income African American women diagnosed with SLE	Self-management; Healthcare Utilization	Participants demonstrated **improvements in** short- and long-term benefits **in patient-centered outcomes and health services utilization** following the intervention. Reported **increased knowledge and confidence** in managing their condition The program was found to be **feasible and acceptable** for this population
2012/ Vina et al. [31]	To determine whether there are racial/ethnic differences in the willingness of patients with SLE to receive *cyclophosphamide* or participate in clinical trials and whether demographic, psychosocial, and clinical characteristics contribute to these differences.	Mixed methods	120 African American and 62 White patients with SLE were evaluated.	Medication Adherence; Trust in Providers	A racial/ethnic variation was associated with belief in medication effectiveness and **trust in the medical provider**,** suggesting that education about therapy and improved trust can influence** decision-making among SLE patients.
2013/ Feldman et al. [23]	To understand the barriers to care faced by women with lupus from medically underserved areas. - To develop interventions to improve care for these women. - To improve lupus education. - To reduce isolation. - To enhance healthcare coordination.	Qualitative Descriptive	29 participants in focus groups/Mean age: 51 years Majority African American (80%); Hispanic (10%) and White (10%)	Satisfaction with Care	The study identified key themes among women with lupus from medically underserved areas, including the need for lupus education, addressing isolation, and overcoming barriers to care. It also identified a need for improved **communication at diagnosis and better navigation assistance** for patients.
2016/Jolly et al. [13]	To evaluate the reliability, validity, responsiveness, and utility of the Lupus Impact Tracker	Mixed Methods; Longitudinal, Cohort observational study	325/90% female, 53% White, 33% AA, Mean Age 42 Years old/	Communication Quality	The **LUPUS Impact Tracker is reliable and valid instrument for assessing the impact of SLE on patients and captures unique and important information not included in physician disease assessments. It may also be useful in a clinical practice setting to facilitate communication** between the physician and the patient and enable efficient incorporation of the patient’s perspective in disease management.
2018/Kasturi et al. [17]	Assess the feasibility of administering the PROMIS computerized adaptative test in Outpatient SLE patients to promote and facilitate patient-provider communication	Quantitative Study design; Observational study, non-randomized, single-site, non-controlled	204 adults aged 18 years or older - Diverse racial and ethnic backgrounds: majority non-white (62.3%), more than one-quarter Hispanic (28.4%) /	Communication Quality and Patient-centered outcomes	Demonstrated feasibility and efficiency of **PROMIS to measure Patient-Centered Outcomes**
2018/ Faith et al. [22]	Examine how AA women with SLE describe their disease experience and how they cope with it.	Qualitative Descriptive; Pre/Post Pilot study/Focus Group	27/Median Age 35–44 adult AA females	Communication Quality; Self-Management; Trust in Providers	Identified Subcategories of themes to include interpersonal relationships, individual experiences, and physician-patient relationships and Highlighted the need for **familial involvement/** **relationship in physician care**,** improved empathy and communication from healthcare providers** and suggesting future interventions should focus on improving **provider sensitivity** and **cultural awareness**.
2019/Qu et al. [14]	To identify factors that might facilitate or impede the implementation of shared decision-making in lupus electronic tool (SMILE) in clinics by assessing perspectives of clinicians, clinic champions, and patient advocacy organization leader	Qualitative Descriptive	23 lupus care providers (18 physicians, 5 champions) and leaders of two patient advocacy organizations	Communication Quality; Shared Decision-Making	Physicians, staff, and patient advocacy **leaders perceived the SMILE as a promising tool to facilitate patient-provider communication and quality improvement** in lupus care. Identifying and addressing patient, clinic-, technology-, and management-related barriers to the SMILE implementation will allow its integration into busy clinical practice workflow.
2019/Twumasi et al. [19]	This qualitative study aimed to explore the perceived impact of a peer-led, group-based educational intervention (the Chronic Disease Self-Management Program [CDSMP]) on healthcare engagement behaviors among African American women with SLE.	Qualitative Descriptive	24 AA/Females, Mean Age 48.6 Years	Communication Quality, Medication adherence, Healthcare Utilization	The CDSMP has the most significant impact on improving communication with doctors. Evidence that **many women in this sample perceived the CDSMP as a valuable resource** to improve healthcare behaviors, including communicating with doctors and managing medication side effects.
2019/Drenkard et al. [1]	Examine whether patient perceptions of physician-patient interactions vary by demographic characteristics, disease activity, and/or depression in African American patients with SLE.	Quantitative Study design; Cross-sectional observational study	698 (80%)/Adult AA, Mean Age 47 with SLE	Communication Quality; Disease Activity/Severity	Identified significant trends of **poorer communication and interpersonal style scores with increasing severity of disease** activity and depression symptoms.
2020/ Rodgers et al. [30]	Examine the impact of the local environment of Charleston, South Carolina on healthcare perceptions and decision-making among patients with SLE, healthcare practitioners, and community members, to inform culturally tailored interventions for different populations.	Qualitative study	Included 19 participants of which 11 were considered AA/Gullah.	Self-Management; Trust in Providers; Health Care Utilization; Shared Decision-Making	This study revealed eleven principal themes affecting healthcare views and decision-making among lupus patients in Charleston, including connectivity, knowledge, and self-management. Research emphasizes that **culturally customized**,** reliable**,** and proficient care is vital** for enhancing results and restoring trust in historically oppressed populations.
2020/ Georgopoulou et al. [11]	To evaluate the impact of physician-patient interaction on medication adherence among patients with lupus nephritis.	Quantitative Study design; Cross-sectional	98 total participants with majority of participants were women (*n* = 84; 85.7%) and English (*n* = 35; 35.5%). Mean age was 40 years (range 21–66; SD 10.94). Forty-four patients (48.9%) had active disease; Exact number of AA is not provided.	Trust in providers, patient participation, communication quality, treatment satisfaction, disease activity, medication adherence; self-management	The findings highlighted two key elements: **(a) the importance of trust in relation to medication adherence and (b) a good understanding of patients’ illness is linked to a better relationship with their doctor and greater participation** in shared decision-making, which is associated with increased trust.
2021/ Plantinga et al. [18]	Assess the comprehension of a novel, individualized functioning report in a predominantly Black SLE patient population. Evaluate the utility of the report for facilitating patient-provider communication about functioning.	Mixed Methods; Observational study; non-controlled; non-randomized; single-site	59 SLE patients; Mean age: 49.6 years; Sex: 91.5% female; Race: 78.7% Black	Communication Quality; Shared Decision Making	The novel functioning report for SLE patients was associated with high comprehension, utility, and acceptability. Most participants found the report easy to understand and useful for care planning. The report had high acceptability for clinical use, with participants feeling comfortable discussing it with healthcare providers.
2021/Sun et al. [3]	To assess the association between patient-provider communication and self-efficacy	Quantitative Descriptive; Cross-sectional observational study	121 patients (37% White, 63% African American),	Communication Quality; Medication Adherence; Self-Management	African American patients with **SLE experience more severe disease and perceive more hurried communication with providers.** Worse communication is associated with lowered self-efficacy.
2021/Singh et al. [16]	Describe the IDEAL study protocol to evaluate the integration of a decision aid for lupus into clinical practice.	Mixed Methods; Observational case study design	Multi-site (15 clinics across the USA); At least 500 patient participants across all sites	Shared Decision- Making, Communication Quality	The primary outcome was the adoption of the decision aid, emphasizing accessibility and usage among qualified patients.
2022/Barr et al. [21]	Identify modifiable factors associated with persistent medication adherence; Explore the relationship between patient-provider communication and patient self-efficacy in the context of medication adherence.	Quantitative Descriptive; Observational study, prospective cohort, longitudinal design	77/Median Age 44, 53% Black; 96% Female	Communication Quality, Medication adherence; Self-Management	Persistent medication non-adherence was associated with patient-perceived **hurried communication (fast speech and difficult vocabulary)** with providers.
2023/ Yalavarthi et al. [24]	To identify opportunities for improving SLE care based on the experiences and perspectives of Black adults with SLE.	Qualitative Study; semi-structured interviews	30 Black adults in Michigan who were diagnosed with SLE, 97% women, Mean age 41;	Communication Quality, Medication adherence; Healthcare Utilization	The study identified four main themes from the experiences of Black adults with SLE: awareness of SLE signs and symptoms, patient-clinician interactions, medication adherence and health effects, and comprehensive care plans after diagnosis. The findings of this study suggest **how experiences of racism**,** limited information about lupus**,** treatment regimens**,** and social risk factors** may affect Black adults with SLE.
2024/ Huot et al. [38]	To create a visual tool to help facilitate the communication of self-management of SLE patients.	Mixed Methods; Observational study; non-controlled; online pilot survey	10 participants completed questionnaires	Communication Quality, Shared Decision Making	The Purple Butterfly nurtures effective doctor-patient communication by **providing concise visual summaries of lupus patients’ health conditions.** Nine out of 10 participants agreed that visuals should be used during medical consultations.
2025/Singh et al. [39]	To assess clinical outcomes, effectiveness and implementation of a computerized lupus patient decision aid (PtDA) in rheumatology clinics.	Mixed Methods; Observational, multi-site, implementation study	Mean age: 44.7 years Sex: 93% female, 6.7% male - Race: 41% White, 45% African American, 8% Hispanic, 4% Asian	Shared Decision Making, Healthcare Utilization	Percentage of patients who viewed the lupus PtDA ranged from 3% to 44% across 15 US rheumatology clinics, indicating variability in implementation success. A higher number of healthcare providers in a clinic were associated with higher penetration rates. Clinical personnel perceived the lupus PtDA as appropriate, acceptable, feasible, successful, and permanent.
2025/Gilman et al. [15]	Describe the underlying methodology of the Strategies to Embrace Living with Lupus Fearlessly (SELF) online digital program. Evaluate the feasibility and acceptability of the SELF program.	Mixed Methods; Observational study; Pilot testing	65% (*n* = 155) completed the initial assessment; Mean age = 45.3;44% White, 23% Black; 15% Hispanic	Self-Management, Communication Quality	SELF is generally feasible and acceptable, particularly for those needing to build lupus self-management skills. Almost 60% of participants who completed the program closed a skill gap by mastering one or more self-management skills. SELF significantly **reduced patient fatigue interference and improved doctor-patient communication**.
2025/ Gunning, et al. [29]	To investigate how women of color with autoimmune diseases deal with and use credibility tactics when they feel disenfranchised by clinician interactions. It also highlights the problems they face when trying to be heard and seen in healthcare settings.	Qualitative study; Observational study; Cross-sectional survey study;	All mono- and multiracial women of color in the U.S. with autoimmunediseases who engage with healthcare clinicians.;The exact number is not Provided.	Community Quality, Psychological distress	The research revealed three innovative credibility strategies to include **contextual racial presentation**,** code-switching**,** and the mobilization of credible witnesses** which are specific to the experiences of women of color with autoimmune disorders. There were two double binds identified to include fracturing identities/fracturing care and shared identities/institutional roles which highlight how racial and ethnic identity can complicate trust between patients and healthcare providers.

**Table 3:** Evidence map of included studies

Domain	Evidence summary
Study designs	Predominantly quantitative (quasi-experimental and cross-sectional), with limited qualitative and mixed-methods studies
Populations	Adults with SLE; limited studies focused specifically on African American/Black patients despite high disease burden
Communication Domain	Shared decision-making, trust, patient-centered communication, health education
Outcomes	Self-management, adherence, patient satisfaction, quality of life
Key gaps	Underrepresentation of African American/Black patients; limited longitudinal and intervention studies; lack of psychometrically validated outcome measures of communication

## Data Availability

The data supporting this literature review’s findings are available upon request from the corresponding author.

## Abbreviations

AA: African American
CINAHL: Cumulative index to nursing and allied health literature
GOAL: Georgians organized against lupus
HRQoL: Health related quality of life
LIT: Lupus index tracker
PRO: Patient reported outcomes
SLE: Systemic lupus erythematosus
US: United States
